# Supplementary material for: Patterns of Intron Gain and Loss in Fungi
Source: PLoS Biol. 2004 Nov 30;2(12):e422. doi: 10.1371/journal.pbio.0020422 (PMC532390; doi:10.1371/journal.pbio.0020422)
Supplement: Table S1 — Also available at http://genes.mit.edu/NielsenEtAl/. (4.3 MB ZIP). [file pbio.0020422.st001.zip › NielsenEtAl/html/854.html]

AN3622.1.NCU08343.1.MG03726.1.FG01503.1


```
 CLUSTAL W (1.82) Multiple Sequence Alignments - Introns Inserted


Sequence 1: NCU08343.1	346 aa
Sequence 2: MG03726.1	348 aa
Sequence 3: FG01503.1	363 aa
Sequence 4: AN3622.1	371 aa
Alignment Length: 381 aa
Number Identitical Residues: 181 aa
Alignment Score (without introns) 8642


MG03726.1 	MADSMSG----TPSPQPDITRDRLPTLFEVLSRRTSPPVDLFSFYIYMRDQQRSVDYLDF
NCU08343.1	MTEPIAH----TPSPQPDLNRNRLPTLFEVLSRRTLPPVDLFSFYIYMRDQQRSVDYLDF
FG01503.1 	MAEHEPVNPMASPSPQPDMTRNRLPTLFEVLSRRTLPPVDLFSFYIYMRDQQRSVDYLDF
AN3622.1  	MT---------TPGMVELEQRNRLPTLFEVLSRRTLAPVDLFSFYIYMRDQQRSVDYLDF
          	*:         :*.      *:************* .***********************

MG03726.1 	W2LDVAQHMTLCRHYVRELRRSVLVGTPDGEKM-SKRSSGILESMGGLDH----GAGPS-
NCU08343.1	W2LDVAQHMSLCRHYVRELRRSVLVGTPEPDRS-SKRSSAILESMGDLNP----VAGPS-
FG01503.1 	W2LDVAQHMSLCRHYVRELRRSVLIGTPEAQ---SKRSSAILENIGDLEPR---AAGPS-
AN3622.1  	W2LDVSQHMSLCRHYVRELRRSVLVATPDLEKAESKGSSTPLENFESVNDIPLVEAGPSG
          	* ***:***:**************:.**: :  .** **  **.: .::  .   ****.

MG03726.1 	-RFGHNTEKTRDQDAQMSAFLRDEASHGIHDSPQSSQSRNARPSG-STPRD-----LTTD
NCU08343.1	-MWAS--EKEKDQDAQMSAFLREDRSI-TQESPQSSTDRRGRPSNLSNQRE-----LTTD
FG01503.1 	-MYAT--EKEKNQDAQMSAFLREDQSH---DSPQSATGPMRPSPQFSNSHD-----ITTE
AN3622.1  	LRHGLRDLDNKEADQRLSAFLRSDGHS-SKHSPQNSLGSQNDPARTMSNEQPRPSLTQHE
          	   .    . :: * ::*****.:      .***.: .    ..   . .:. .:    :

MG03726.1 	SNSPMHAVAREDIRASAEKILYTFLLPGAEREIVLPGSITQDVTTAIEEFGRDDPEGFDV
NCU08343.1	SNSPAHAVARDDIRASAEKILYTFLLPGAEREITLPGSITSDVTTAIEDYGRDDPEVFDV
FG01503.1 	SNSPAHTVARQDIRASAEKILYTFLLPGAEREITLPGSITQEVTAAIEEYGRDDPEVFDV
AN3622.1  	SSSPGHTVARGDIRASAEKILYTYLLPGAEREIVLPEEMVSSIINLVEDDGRDDPEVFDP
          	*.** *:*** ************:*********.** .:...:   :*: ****** ** 

MG03726.1 	AKDYVFQAMERDAFPGFLRMKALGNLIPPTLIMRLILGMVSMFGGFWAAFVLIFLNTPWQ
NCU08343.1	AKDYVFQAMERDAFPGFLRMKALGNLIPPTLMLRLMIGLVAMFGGFWTAFTLIFLNESRQ
FG01503.1 	AKDYVFQAMERDAFPGFLRMKALGNLIPPTLIMRLILGLISMFAALWASFVLIFLDYSRT
AN3622.1  	AKDYVFQAMERDAFPGFLQAKALGNLVPLSIMARLAFALISFGGGFWGAFYVVLRDKPRN
          	******************: ******:* ::: ** :.:::: ..:* :* ::: : .  

MG03726.1 	VRLW0----------------YSLDPILALLGFSEYTPFNFSRVREPYVRKLLAKRSIMV
NCU08343.1	TRCW0----------------YSLDPILALVGLSEYTPFNFSRVREPYVRRLLAQRSMVV
FG01503.1 	TRCW0LILPFTIGVYFLASYQYSLDPIMALIGYSEYTPFNFSRIREPYVRKLIAKRAMMV
AN3622.1  	IRCW0VILPFVIACYFIVSYQYKIDPVMAFAGYSEYTFMNWSPVREPYVRKLLVKRAVAT
          	 * *    .   .     : .*.:**::*: * **** :*:* :******:*:.:*:: .

MG03726.1 	LATTILVDAALLALFIAVPGKRL
NCU08343.1	LAVTAVVDAALCVLFILVPGKRL
FG01503.1 	MAVTFLVDAALCVLFILVPGKRL
AN3622.1  	VLIASFVAAALSILFILVPGTML
          	:  : .* ***  *** ***. *
```
